# Development towards Compact Nitrocellulose-Based Interferometric Biochips for Dry Eye MMP9 Label-Free In-Situ Diagnosis

**DOI:** 10.3390/s17051158

**Published:** 2017-05-19

**Authors:** Beatriz Santamaría, María F. Laguna, David López-Romero, Ana L. Hernandez, Francisco J. Sanza, Álvaro Lavín, Rafael Casquel, María V. Maigler, Rocío L. Espinosa, Miguel Holgado

**Affiliations:** 1 Centre for Biomedical Technology, Optics, Photonics and Biophotinics Laboratory, Campus Montegancedo, Universidad Politécnica de Madrid, 28223 Madrid, Spain; beatriz.santamaria@ctb.upm.es (B.S.); ana.lopez@ctb.upm.es (A.L.H.); alavin@etsii.upm.es (A.L.); rafael.casquel@ctb.upm.es (R.C.); rocio.lopez@upm.es (R.L.E.); m.holgado@upm.es (M.H.); 2 BioOptical Detection, Centro de Empresas, Campus Montegancedo, 28223 Madrid, Spain; d.lopez-romero@biod.es (D.L.-R.); fj.sanza@biod.es (F.J.S.); m.maigler@biod.es (M.V.M.)

**Keywords:** nitrocellulose, biosensor, photonic, label-free, dry eye, MMP9, low detection, low volume, compact, biosurface

## Abstract

A novel compact optical biochip based on a thin layer-sensing surface of nitrocellulose is used for in-situ label-free detection of metalloproteinase (MMP9) related to dry eye disease. In this article, a new integrated chip with different interferometric transducers layout with an optimal sensing surface is reported for the first time. We demonstrate that specific antibodies can be immobilized onto these transducers with a very low volume of sample and with good orientation. Many sensing transducers constitute the presented biochip in order to yield statistical data and stability in the acquired measurements. As a result, we report the recognition curve for pure recombinant MMP9, tests of model tears with MMP9, and real tear performance from patients, with a promising limit of detection.

## 1. Introduction

In 1992, the World Health Organization published a relevant classification of diseases and injuries related to the eye. These known eye diseases have proliferated in the last decade probably because of the use of information and communication technology-based devices such as computers and smart mobiles. Dry eye syndrome (DES) causes irritation, stinging, redness, and fatigue, among others—early detection and treatment being essential for the prevention of eye damage. Unfortunately, an effective correlation between signs and symptoms is not always clear, and a reliable in-situ diagnosis of DES is strongly recommended. In this term, several and relevant biomarkers are being validated to dry eye diagnosis [1]: MMP9, CST4, S100A6, ANXA1, ANXA11, and PLAA. However, despite the promising accurate diagnosis that can be obtained from these biomarkers, in-situ diagnostics needs quantitative results by using a limited volume of tear sample.

The compact biochip reported in this article faces these two barriers: obtaining quantitative information with a limited volume of sample. Although each of the above-mentioned biomarkers can be monitored with this compact biochip, in this article, we report, as a proof-of-concept, the detection of an MMP9 biomarker. The reason is because MMP9 is the most challenging in terms of the limit of detection and is the most reported in the literature, and there are commercially available model tears to compare the results.

In 2016, Lanza and co-workers reported a worthy point-of-care device for testing MMP9 with a limit of detection (*LoD*) of 40 ng/mL [2]. However, according to the results for MMP9 reported in patients with ocular surface disease, several authors observed that an MMP9 concentration range between 6 and 40 ng/mL (average 23.61 ng/mL) can be considered as a control, whereas concentrations from 47 to 146 ng/mL (average 97 ng/mL) can be considered DES [3], according to the protocol of clinical diagnosis [4,5].

In previous works, we reported biophotonic sensing cells (BICELLs) as an efficient methodology for label-free detection of antibodies in purified samples [6,7]. In this label-free biosensor, the sensing response is determined by the optical interferometric signal produced when the specific antibodies capture the target biomarker without any chemical amplification and labels.

In this article, we evaluate a new biochip with three integrated interferometric transducers (BICELLs) where the novelty of using a thin layer of nitrocellulose as a sensing surface allows one to significantly enhance the immobilization process of the MMP9 antibody. As a result, an efficient recognition of the MMP9 antigen is directly detected without any added labels. We report in this work, the recognition curve of MMP9 biomarker and study the response of model samples and real tears on this surface. The detection procedure is performed directly by a label-free in vitro diagnosis (IVD) with a single drop of tear and a low volume of sample needed. The results demonstrate that a lower concentration of MMP9 in the tear model matrix can be detected and assess the feasibility for in-situ IVD of dry eye disease in real tears.

## 2. Materials and Methods 

### 2.1. BICELL Fabrication and Materials

We integrated three interferometric BICELL transducers in a single biochip, fabricated by two interferometric layers, SiO_2_ and SU8 resist, plus a thin layer of nitrocellulose (Sigma-Aldrich, St. Louis, MO, USA) per biochip. The manufacturing was carried out at the wafer level [8], where the difference with previous reported fabrication is the deposition of the nitrocellulose by spin coating over the SU8 surface [9]. 

**Figure 1:**
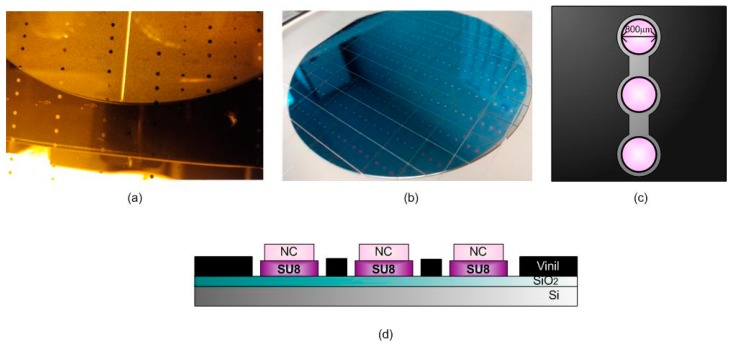
(**a**) Photo mask; (**b**) sensing surface at wafer level; (**c**) outline of each chip with three sensing cells; (**d**) transverse view of a single transducer and the forming layers.

The wafer is overlaid by SU8, and it is exposed to a thermal treatment and, after the photoresist, is cured by optical contact lithography with mask. Finally, another thermal treatment is performed, and the wafer is developed to remove remaining resist [10]. The soda lime glass mask with a chrome layer used in the lithography (manufactured by JD Photo Data, Herts, UK) was designed to integrate the three BICELL transducers 800 μm in diameter in a single biochip (Figure 1). To create the last layer of the biosensor, the nitrocellulose is spun over the SU8 cells and exposed by DUV with a quartz mask inversely copied from the mask used in the first lithography step. After that, the remains are eliminated with a PMMA industrial developer AR-600-55 (ALLRESIST GmbH, Strausberg, Germany) diluted 1:4 with propanol. When the BICELLs were manufactured chip by chip, the cleaning process was exhaustive [11]. However, fabrication at the wafer level drastically improves the yield because the cleaning process is reduced in a single rising step with dimethyl sulfoxide (DMSO).

Finally, the fluidic structure with the three wells where dropping the tears are based on vinyl, forming the integrated biochip with three BICELLs as shown in Figure 1. The combination of both nitrocellulose-based transducers and a fluidic structure allows for a low volume of sample usage.

### 2.2. Optical Characterization of the Biosensor

As mentioned above, the biochip layout has three BICELLs as biotransducers. The optical response of the BICELLs changes due to the refractive index variation caused by the immobilization processes (bioreceptors) and the recognition events (MMP9 detection). In order to read-out each BICELL signal response, a Fourier Transform Visible-Infrared (FT-VIS-IR) spectrometer (Bruker Vertex 70 adapted to visible range) [12,13] is employed. This optical tool is capable of focusing on each BICELL through an optical objective with a magnification of 4× at normal incidence capturing the interference spectrum (reflectivity as a function of wavelength) with a 1 cm^−1^ resolution. 

### 2.3. Biofunctionalization of the BICELLs 

In order to detect the MMP9 protein (Sino Biological Inc., Beijing, China), a direct immunoassay was performed. 

Firstly, the sensing surface needs to be washed with Milli-Q water. Thus, the dust and other particles from the fabrication and storage are easily removed. Moreover, this washing step helps to nitrocellulose surface activation, and it becomes an optimum layer for binding the biological molecules electrostatically. A biofilm of protein A (Sigma-Aldrich) is bound to the sensing surface of each BICELL of the biochip. The immobilization of protein A takes place incubating 50 μg/mL in phosphate-buffered saline (PBS). For this immobilization, only a volume of 3 μL of protein solution is incubated in each transducer for 30 min at 37 °C in a humid atmosphere. The washing step is done with 5 mL of distilled water and is dried with clean, dry, dust-less air. The wavenumber shift observed for the protein A immobilization was 20 ± 5 cm^−1^. 

Then, an incubation of anti-MMP9 (Sino Biological Inc.) antibody is carried out to properly immobilize and orientate the anti-MMP9 onto this sensing surface. Protein A helps to bind the antibody in a desired orientation for a better recognition of the antigen. The antibodies used were previously tested by ELISA, and the affinity between antibody-antigen was ensured. Thus, 3 μL of volume is incubated for 14 h at 37 °C in a humid atmosphere of anti-MMP9 at a concentration of 50 μg/mL in PBS. The washing for this step is with 20 mL of PBT-Tween and 10 mL of distilled water. The wavenumber shift observed for the protein A + Anti-MMP9 biofilm was 95 ± 5 cm^−1^. BICELLs out of this tolerance range were rejected and not considered for the recognition assays.

Lastly, we proceeded with the sensing surface blocking step to avoid unspecific binding, leading one of the most important steps for the specific optimal MMP9 recognition. For this purpose, a bovine serum albumin (BSA) (Sigma-Aldrich) blocking agent was used for 1 h of incubation at 37 °C in a humid atmosphere. The volume of this solution in each BICELL was also 3 μL. The washing step was the same as that previous described for the antibody immobilization, but it needs 60 mL of PBS-Tween and 30 mL of distilled water. We also checked that both protein A and the BSA blocking agent used did not offer significant specific affinity reaction to MMP9.

Once each of the BICELLs had the anti-MMP9 immobilized onto the sensing surface and blocked, MMP9 antigen was specifically recognized, as reported in the following sections of this paper. 

## 3. Results 

### 3.1. Recognition Curve Response to Detect MMP9

**Figure 2:**
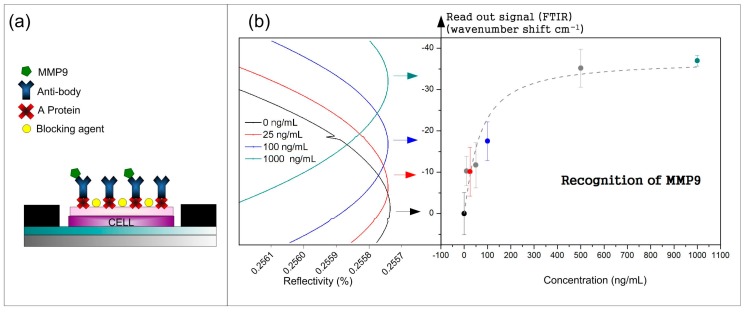
(**a**) Diagram of a direct immunoassay and (**b**) recognition of MMP9: wavenumber shift monitored in function of concentration and reflectivity.

Following the biofunctionalization process, the recognition of the MMP9 recombinant protein is performed. The recombinant protein is detected in an accumulative direct immunoassay in 3 kits. Per each BICELL on the integrated biochip, 3 μL with increasing concentrations (10, 25, 50, 100, 500, and 1000 ng/mL) were incubated for 20 min at 37 °C. The increases were carried out with 5 mL of distilled water. Each concentration signal is monitored through the average wavelength shift of a minimum of interference of the three BICELLs. Monitoring this wavelength shift (readout signal) as a function of concentration allows for the recognition of the MMP9 sensing response. Figure 2 shows the wavelength shift of the interferometric signals and the recognition curve of the MMP9.

The sensing response obtained for the MMP9 allows us to determinate the limit of detection (*LoD*). According to previous works [14], the *LoD* can be estimated by the quotient between the expanded uncertainty (*U*) and the sensitivity (*m*): *LoD = U/m*. In order to calculate the expanded uncertainty, a coverage factor of 3 (*U = 3u*) is used, as this is recommended by the International Union of Pure and Applied Chemistry (IUPAC), where *u* is the typical uncertainty. The statistical standard deviation (*s*) used to be the only uncertainty factor to consider in many articles. In other articles, only the instrument resolution is taken into account. However, for this type of sensor, both instrument resolution and statistical standard deviation have to be borne in mind. Considering the linear part of Figure 2b and the expanded uncertainty, the estimated *LoD* value was 25 ng/mL. 

### 3.2. Recognition of Model Samples

The human tear is a fluid with a mixture of proteins, glycoproteins, lipids, small molecules, etc. A healthy tear is composed of nutrients, salt electrolytes, antimicrobial molecules, peptides, and many other components that interfere with the measure of MMP9 [15,16]. Due to this matrix, model samples are measured to verify the affinity between immobilized antibody and antigen (MMP9). Used contrived samples from Ursa BioScience Company are composed of NaCl, KCl, sodium bicarbonate, urea, ammonia chloride, γ-globulins, vitamin C, citric acid, albumins, lysozymes, pyriuvic acid, lactic acid, and hidrochloric acid, but only by MMP9 protein in different concentrations [17]. 

**Figure 3:**
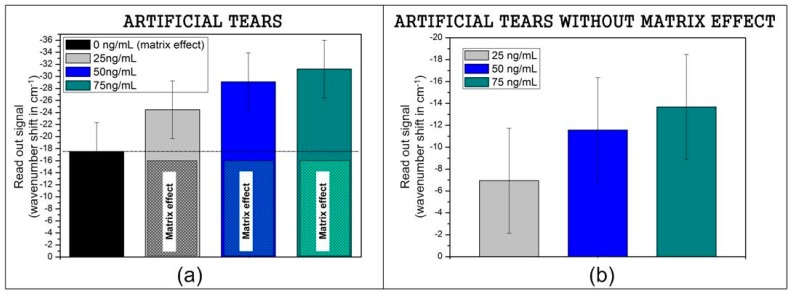
(**a**) Raw signal of MMP9 in contrived tears; (**b**) MMP9 signal in contrived tears subtracting the matrix effect.

The same biofunctionalization protocol employed for a recombinant protein is used for the model samples. For the recognition instead, a different concentration is incubated on different biosensing surfaces: 0, 25, 50, and 75 ng/mL. The volume used was also 3 μL per each BICELL of the integrated biochip for 20 min at 37 °C and is washed with 5 mL of distilled water. The first incubation (with no protein) shows an increased readout signal due to a matrix effect that is not avoided with the blocking process. This signal is considered the background to remove from the raw sensing readout concentration signal, as shown in Figure 3. The signal increases with the concentration of MMP9 in the model matrix. It is possible to determinate the dynamic range for the artificial tears. This method detects a concentration from 25 ng/mL, the average value for a control eye, until 75 ng/mL (30 cm^−1^ of wavenumber shift according to the saturation in Figure 2b).

### 3.3. Recognition of Real Tears

Additionally, a preliminary study of real tear samples was completed. Tears considered in this assay were from three patients classified as control eye and three patients as dry eye supplied by BIOFTALMIK S.L. (Three kits used per pathology means nine replicates measured). The samples were collected without stimulation by capillary tubes to avoid a non-desired tear dilution caused by the irritation, stress, or direct contact of stimulated collection [18]. Then tears were stored at −80 °C until analysis. The recognition protocol was carried out in the same manner as done with the recombinant protein and contrived tears: 3 μL of sample volume per cell, similar time and same temperature during the incubation. For signal processing the matrix effect obtained in Figure 3 was considered.

The results related to real tears show differences between control eye and dry eye patients. The media of the signal obtained for three control eye samples (1 kit per patient) was 5.6 cm^−1^, in good agreement with the signal obtained for 25 ng/mL of artificial tears (6.9 cm^−1^) as shown in Figure 3, and it corresponds with the values for control eye. 

**Figure 4:**
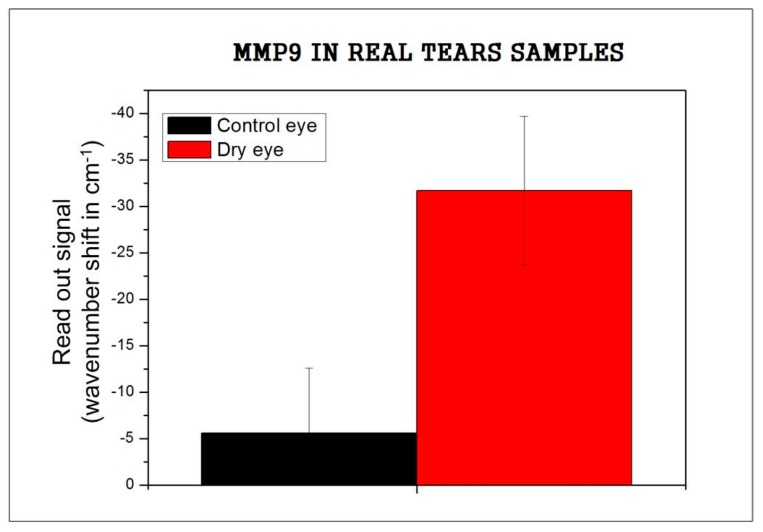
MMP9 signal in real tear samples.

Comparing the signal for three dry eye samples (31.7 cm^−1^), with the results obtained with the artificial tears, the signal was higher than 75 ng/mL (13.7 cm^−1^ for model samples), which concurs with the dry eye diagnostic (Figure 4). 

## 4. Discussion

A new biochip with three integrated interferometric sensing cell transducers, BICELLs, based on nitrocellulose sensing surface for in-situ and IVD label-free detection of MMP9 was fabricated. The proposed biochip has the capability of integrating three BICELLs, where antibodies were immobilized with good orientation thanks to the previous step of protein A adsortion onto the nitrocellulose sensing surface. 

Although the recognition curve of the recombinant protein began with a solution of 10 ng/mL, the differences between concentrations started to appreciate in a concentration of 25 ng/mL. This result concurs beside the signals obtained with model samples, where the matrix effect is evaluated for the null MMP9 concetration (0 ng/mL) signal and substracted from other concentrations. 

Finally, a preliminary report of real tears is tested and variation between control eyes and dry eyes can be noticed. 

## 5. Conclusions

In this article, a new integrated biochip for detecting low concentrations of MMP9 with a suitable recognition sensing curve response is reported for the first time. The presented work shows a quantification detection of MMP9 in different recombinant protein solutions. Moreover, artificial tears with known MMP9 concentrations measured allowed for an evaluation of the matrix effect. Finally, two real samples from patients (control eye and dry eye) were tested, and a qualitative study was performed. 

It should also be noted that the low volume of antibodies and tears needed to carry out the assays improves the cost-effectiveness of the compact biochips. 

As the results, we can account a limit of detection of 25 ng/mL with this type of biochip on the recombinant protein and its ability to monitor real tears in a very small volume of sample. Additionally, regarding the qualitative study of tears, in the future, increasing the number of measurements and improving the instrument resolution can enable a quantitative test. Moreover, these results open the possibility of screening tear biomarkers directly and in-situ at the point-of-care level.
